# A real-time medical cartography of epidemic disease (Nodding syndrome) using village-based lay mHealth reporters

**DOI:** 10.1371/journal.pntd.0006588

**Published:** 2018-06-15

**Authors:** Raquel Valdes Angues, Austen Suits, Valerie S. Palmer, Caesar Okot, Robert A. Okot, Concy Atonywalo, Suzanne K. Gazda, David L. Kitara, Moka Lantum, Peter S. Spencer

**Affiliations:** 1 Department of Neurology, Oregon Health & Science University, Portland, Oregon, United States of America; 2 University of Washington, Seattle, Washington, United States of America; 3 Hope for Humans, Gulu, Uganda; 4 Awere Health Center III, Pader, Uganda; 5 School of Medicine, Gulu University, Gulu, Uganda; 6 MicroClinic Technologies, Ltd, Nairobi, Kenya; University of California San Diego School of Medicine, UNITED STATES

## Abstract

**Background:**

Disease surveillance in rural regions of many countries is poor, such that prolonged delays (months) may intervene between appearance of disease and its recognition by public health authorities. For infectious disorders, delayed recognition and intervention enables uncontrolled disease spread. We tested the feasibility in northern Uganda of developing real-time, village-based health surveillance of an epidemic of Nodding syndrome (NS) using software-programmed smartphones operated by minimally trained lay mHealth reporters.

**Methodology and principal findings:**

We used a customized data collection platform (*Magpi*) that uses mobile phones and real-time cloud-based storage with global positioning system coordinates and time stamping. Pilot studies on sleep behavior of U.S. and Ugandan medical students identified and resolved *Magpi*-programmed cell phone issues. Thereafter, we deployed *Magpi* in combination with a lay-operator network of eight mHealth reporters to develop a real-time electronic map of child health, injury and illness relating to NS in rural northern Uganda. Surveillance data were collected for three consecutive months from 10 villages heavily affected by NS. Overall, a total of 240 NS-affected households and an average of 326 children with NS, representing 30 households and approximately 40 NS children per mHealth reporter, were monitored every week by the lay mHealth team. Data submitted for analysis in the USA and Uganda remotely pinpointed the household location and number of NS deaths, injuries, newly reported cases of head nodding (n = 22), and the presence or absence of anti-seizure medication.

**Conclusions and significance:**

This study demonstrates the feasibility of using lay mHealth workers to develop a real-time cartography of epidemic disease in remote rural villages that can facilitate and steer clinical, educational and research interventions in a timely manner.

## Author summary

Absence of health monitoring of rural populations in low-income countries allows diseases to emerge and spread for months before their detection by public health authorities. We tested the feasibility of using smartphones operated by lay villagers to report health information in real time from the populations in which they reside. Eight young lay adults from remote rural regions of northern Uganda were trained to administer questions and transmit answers using pre-programmed mobile phones. Weekly, over a 3-month period, each lay reporter monitored an average of 40 children suffering from an epileptic disorder known as Nodding syndrome (NS). For each child, episodes of head nodding, convulsions, injuries, deaths and availability of anti-seizure medication were reported weekly and the data instantaneously assembled by customized software for analysis in Uganda and the USA. This system not only provided a real-time map of the health status of children with established NS but also discovered children previously unknown to have head nodding. While logistical hurdles had to be overcome, the study demonstrates the feasibility of using lay workers operating software-equipped mobile smartphones to build a current and continuously updatable medical geography of the rural populations in which they reside. Wide application of such systems could result in the early detection and control of disease.

## Introduction

Disease outbreaks in remote rural populations of Africa and elsewhere are often detected late by public health entities. Reasons include dependence on traditional remedies and healers, low village literacy and knowledge of how to report a disease outbreak, lack of distributed health professionals, poor communication systems and difficulties in transportation of patients to clinics. Examples abound but come into focus most dramatically from two outbreaks of Ebola hemorrhagic fever (EHF) in sub-Saharan countries. In an example from northern Uganda (Acholiland), several weeks elapsed between the presumptive index case (August 30, 2000) and virus confirmation (October 15, 2000) of an outbreak of EHF that persisted for 6 months (January 9, 2001) and resulted in 425 presumptive case patients [1]. Second, a major 2014–2016 West African EHF epidemic appears to have begun in rural southeastern Guinea but months elapsed before the illness was recognized as EHF, during which the virus spread to multiple neighboring countries. As of June 2016, 28,616 suspected, probable, and confirmed cases with a total of 11,310 deaths were recorded in Guinea, Liberia, and Sierra Leone [2–3].

These examples illustrate the urgent need to develop improved health surveillance of remote rural communities for emerging and extant diseases. Timely discovery of outbreaks cannot be accomplished by periodic visits by healthcare workers to the scattered villages where disease can begin and spread. Needed is a health surveillance system that in real-time can pinpoint and track disease cases longitudinally, such that clinical, education and research resources can be rapidly and efficiently dispatched to affected villages. To this end, we have tested the feasibility of using software-coupled mobile phones operated with minimal training by village-based lay mHealth reporters charged with repeatedly assessing the status of children with a non-infectious and easily recognized idiopathic neurologic disease (Nodding syndrome) [4–5]. Their weekly reports, transmitted by simple smartphones, were instantaneously aggregated, analyzed and mapped by data collection centers locally (Gulu, Uganda) and remotely (Oregon, USA). While lay health aides have been used successfully to amplify health coverage in remote regions of Asia (Nepal, Bangladesh, Indonesia) [6–9], and mHealth projects are legion [10–12], we are unaware of prior initiatives that have used the reports of lay mHealth operators to populate a real-time medical cartography that can guide clinical, research, and educational interventions to respond to epidemic disease.

## Methods

### Objective

We undertook a feasibility study in remote regions of northern Uganda to determine whether minimally trained lay reporters, resident within rural villages, could collect and reliably transmit health-related information at regular intervals using simple smart phones equipped with network-linked software that integrates data sets from multiple settings across time. The study was not designed or approved to conduct or implement a medical intervention.

### Study setting and participants

This study was conducted in Acholiland, the northernmost region of Uganda. The region is recovering from a brutal conflict (1986–2009) between the Lord’s Resistance Army and the forces of the Ugandan government, which from approximately 1996 to 2008 required an estimated 2 million people to leave their villages for the relative safety of internal displacement camps.

Focus was placed on families with children with Nodding syndrome (NS), a chronic epileptic encephalopathy of unknown etiology that was regionally epidemic between 1997 and 2012, with peaks in 2003–2005 and 2008, 5–6 years after peaks in the number of wartime conflicts and deaths. The largest number of NS cases followed 5–7 years after the peak of population translocation to internal displacement camps [13].

Selected for study were 2 rural districts heavily affected by NS, namely Pader and Omoro, including 2 sub-counties, 4 parishes and 10 villages therein (Table 1). A manageable sample of households (n = 240) with one or more children with NS was recruited in equal proportion for each district. Most if not all children had a prior physician-diagnosis of NS that allowed them to receive, under the treatment program of the Ugandan government [14], a supply of nutritional supplements (Mamas Nutritional Supplement Ltd, Mbale, Uganda) and anticonvulsant medication.

**Table 1:** Districts, sub-counties, parishes and villages participating in the mHealth-based community surveillance study.

**District**	Pader	Pader	Omoro	Omoro
**Sub-County**	Awere	Awere	Odek	Odek
**Parish**	Bolo	Angole	Lamola	Palaro
**Village**	Bolo Juklebi Bolo Agweng Bolo Lapeta	Atede West Paikat Akidi	Akoyo Ajan	Ludok Lukee Olam

A medical diagnostician (DLK) physically examined a sample of households reporting existing and newly reported NS cases, the large majority of which had longstanding but unregistered NS. In agreement with the First International Scientific Meeting of NS in Kampala (July 30^th^-August 1^st^ 2012), a probable NS case was identified by an age of onset between 3 and 18 years of age, a frequency on unprovoked head nodding of 5-20/minute (triggered by food and/or cold weather) and at least one of the following minor criteria: a) other neurological abnormalities (other seizures, cognitive decline/behavioral problems with or without school dropout, psychiatric symptoms), b) developmental abnormalities (stunting or wasting, delayed sexual or physical development) and/or c) clustering in space or time with similar cases.

### Study design

In undertaking this feasibility study, we customized a data collection platform named *Magpi* that uses mobile phones and real-time cloud-based storage with global positioning system (GPS) coordinates and time stamping [15]. The decision to use *Magpi* was based on several factors, including its widespread acceptance (*Magpi* has been used by organizations such as the WHO, CDC, UNICEF, UNFPA, CARE and the IFRC), proven track record and because the software supports: a) a wide variety of question types (text/numeric entry, multiple choice, etc.); b) customized questions; c) off-line data collection (data can be collected off-line and submitted to a central server whenever an active connection becomes available); d) GPS stamping and display of data-points on an electronic map; and e) instantaneous data integration from multiple users. In addition, *Magpi* is f) user friendly; g) easy to learn; h) requires no programing experience (the software allows non-technically trained users to create data collection forms, messaging systems and interactive reports); and i) offers free and paid premium versions with an excellent technical support team. A pilot study in the USA used *Magpi*-programmed basic mobile flip phones, while the pilot and feasibility studies in Uganda used similarly programmed smartphones. The two pilot studies (USA and Uganda) were conducted using the free version of *Magpi* whereas we used the *Magpi* Pro version ($500/month or $417/month if bought annually) to operate the feasibility study in Uganda, which required more than 500 survey uploads/storage per month.

### Pilot mHealth studies (monitoring sleep behavior)

Technical and subject-use issues with *Magpi* software-enabled equipment were identified by conducting two pilot studies among: a) university students in the USA Pacific Northwest and b) students attending Uganda’s Gulu University (GU) School of Medicine. Briefly, each student (n = 12 per study population, with equal numbers of males and females of similar age randomly selected at each site) was provided with a *Magpi*-programmed cell phone, instructed on its functions, and asked to respond to a 10-question personal sleep-health survey daily for 14 consecutive days. After completion, participants were asked to attend a final meeting, share their experiences, return their cell phones, and receive a vendor gift card or small compensation.

While not designed to conduct a scientifically defensible survey of sleep patterns, the data were analyzed using Microsoft *Excel* to assess sleeping trends across the 14-day span. Minor technical difficulties (duplicate questions, duplicate surveys, error messages, etc.) were referred to *Magpi*’s technical support team, and modifications were made to the software feature to ensure these issues were eliminated. The pilot studies also provided valuable information on network connectivity and reporter reliability, perception, and experience in using the application and surveys. Such information fed into the design of the mHealth feasibility study in Uganda.

### Feasibility mHealth study (Nodding syndrome)

The study was carried out in northern Uganda from July to October 2017. Eight male and female Ugandan lay mHealth reporters, age 20–30 years, were recruited and trained. The training covered basic concepts of health and disease, the purpose and content of the program, clinical and social aspects of NS, use and maintenance of the equipment (cell phones, solar batteries, bicycles), questionnaire presentation, data collection and submission, home and community entry, informed consent administration and patient confidentiality. Focus was placed on some typical mistakes, such as mistyping or duplicated forms; time spent collecting and transmitting the surveys; WiFi and Internet accessibility; GPS stamping; and other technical issues such as how to turn off/on the WiFi/GPS to help improve cell-phone battery life.

One local field coordinator and two community health nurses (one from each sub-county), in the role of field supervisors, were appointed and registered with the study to closely monitor the training and operations in the field and to provide onsite supervision to each sub-county mHealth team (4 reporters per sub-county). mHealth reporters were pilot-tested and evaluated for satisfactory performance before they returned to their respective villages to commence weekly reporting. Before engaging in data collection, the mHealth reporters signed the *Hope for Human*s’ Child Protection protocol to ensure adherence with their Child Protection Policy and information confidentiality. Supportive supervision was made available to mHealth reporters by phone (24/7) and in-person every week during the first month of program implementation and biweekly throughout the end of the program. Biweekly meetings of the lay mHealth operators and field supervisors allowed mHealth staff to share field experiences, lessons learned, challenges, barriers and how they could be overcome.

Payment of mHealth reporters was made in the form of a stipend for training, a regular monthly salary, and small economic incentives. For instance, provided weekly mHealth data were uploaded on time to the *Magpi* server, we placed no restrictions on the phones regarding the apps that could be accessed/installed or the use of airtime for personal communication. The phones were loaded with a shared bundle (Kazi, MTN Service Center, Gulu; approx. $97.1/month) from the local mobile operator MTN which included MTN minutes, minutes to other networks, MTN SMS, SMS to other networks, data/internet, and free calling among each number in the bundle.

### Equipment

Inexpensive, locally purchased airtime-loaded smartphones (MTN Smart Mega; approx. $30/phone) supported by the local telecommunication company MTN, were individually programmed with *Magpi* software over 1 week and distributed to the mHealth reporters and field supervisors. With every phone, a portable power supply consisting of a solar energy panel/bulb and a battery with dual USB charging ports (approx. $30/set) was also provided. Paper forms were distributed as a backup alternative to mHealth data collection (e.g. in case of cell phone loss or failure). In addition, mountain bicycles (Phoenix single frame, approx. $80/bike) and raingear (raincoats and boots, approx. $11/set) were supplied to facilitate access and travel to and within the communities (typically with poor or no road infrastructure, particularly during the rainy season). Reserve equipment (two software-equipped smartphones, two solar panels and chargers, and two bicycles) was also available for immediate use in both sub-counties.

### Survey development and data collection

A simple structured questionnaire was designed using the *Magpi* web-interface to measure the feasibility and practicalities of the *Magpi* application-based reporting system. The *Magpi* application works with the *Magpi* website to deploy data collection surveys to basic smartphones. The survey, which was applied once weekly, included information on coded household location, number of NS children/household, seizure frequency, availability of anti-seizure medication, and injuries and deaths.

After obtaining informed consent from the participant household caregivers, a unique ID code was assigned to each household to preserve confidentiality and anonymity. Data collection via the *Magpi’s* mobile platform took place for 12 consecutive weeks, from August 1^st^ throughout October 31^st^ 2017. During this period, the mHealth reporters were asked first to complete and store the surveys locally on their mobile devices in the field, and subsequently, depending on WiFi availability, upload them daily or weekly to a secure web-hosted database.

Day-to-day technical support was provided by the local field coordinator and supervisors; support included: troubleshooting the phone system, applications, survey forms and dealing with any queries from the mHealth workers. Any issues the local research team could not resolve were passed to the program manager at OHSU for investigation.

### Data analysis and monitoring

Online software-integrated village-based survey forms, transmitted by smart phone to the *Magpi* data management website, were monitored weekly and analyzed by the program manager (RVA) located at Oregon Health & Science University (OHSU). Data were later imported into a local data center established at Gulu University (GU) School of Medicine.

During the 12-week data collection period, any mistyping, duplicated reports, data inconsistencies or doubtful information were reported by phone or email to the field coordinator and from there to the corresponding field supervisor for double-checking, fixing, and resending if necessary. On-site monitoring trips were also organized biweekly during the implementation phase to some randomly chosen households, to ensure compliance with data transmission instructions and consistency across different sites and different data collectors.

While the goal of this intervention was to test the feasibility of NS-related data collection and integration using *Magpi*, cartographic information from the mHealth syndromic surveillance within the study area was offered to supervisors and health bureaus in support of their public mandate and with the aim of increasing understanding on how convulsive disorders, such as NS, impact families and children. These data were analyzed qualitatively (using *Magpi* and *Excel*) and reports were mainly descriptive.

### Data security

All cell phones were password-protected, and the survey was developed to avoid subject-specific identifiable information. As mentioned earlier, each household was assigned a specific identification code and each reporter was given a unique identification email. Hence, the data collected and transmitted daily did not disclose information about specific individuals.

All connections between the cell phone and the server were made using modern encryption methods to transfer data, including Extended Validation Secure Sockets Layer (EV-SSL) and AES-256 bit encryption. In addition, *Magpi* uses Rackspace Cloud Storage in the U.S. for primary data storage, which has a robust security policy of its own. Access to the surveys and databases was strictly controlled and only available to authorized personnel. Administrative rights to view data were given to the leadership at GU. Neither the analytics dashboard nor the mobile phone forms gave users any access to edit or change data. This ensured data integrity and eliminated the possibility of data tampering.

### Evaluation

A secondary goal of this project was to a) incentivize and empower young adults with modest education to raise awareness in their communities of the importance of healthcare, disease early detection and disease monitoring, and b) expose GU medical students to community-specific health challenges. The interaction of mHealth reporters and GU medical students with members of the USA team, GU faculty, and health personnel at the HfH care facility, was expected to raise their interest in pursuing and/or expanding upon a health-sector career, whether as a research assistant, nurse, or community health worker, thereby helping to alleviate the health-manpower shortage, building capacity and strengthening a weak primary health care system.

Hence, at the end of the program, we evaluated with short questionnaires the impact of this exploratory mHealth study on mHealth reporters (future healthcare interest), medical students (future research interest), and health workers (acceptability of mHealth and eHealth approaches and compatibility with regional/national systems). As part of the qualitative evaluation, we sought to elicit the views of participants on their acceptability of the intervention and research procedures, as well as potential demand and integration into primary healthcare settings.

### Study approval and consent

The Ugandan study was reviewed and approved by the Institutional Review Board of St. Mary’s Lacor Hospital in support of Gulu University, the Uganda National Council for Science and Technology, and the Office of the President of Uganda. The USA and Uganda studies were reviewed and approved by the Institutional Review Board of Oregon Health & Science University.

Written or fingerprinted consent for participation was obtained for each household, and families were informed about their right to withdraw from the study at any time. At the end of the study, each household received financial compensation ($10) for their time and cooperation.

## Results

### Pilot mHealth studies (monitoring sleep behavior)

We used two pilot studies to test the feasibility of using customized network-linked software (*Magpi*) that integrates data sets from multiple geographically dispersed reporters via mobile phones. The first pilot study employed 12 students from Oregon Health & Science University (Portland, OR) and Washington University (Seattle, WA) as reporters of their own sleep-related behaviors. We used *Magpi*-programmed flip phones to collect and integrate data, develop a real-time sleep pattern topography, and test the hypothesis that 85% of daily sleep surveys, over a 14-day span, would be completed on time. We reproduced the pilot study in Gulu, northern Uganda, with 12 medical students from Gulu University (GU) and basic smartphones locally purchased and supported by the local telecommunication company MTN. Overall, respondents in the USA completed a mean of 77% of surveys administered, compared to a 96% survey completion rate for Ugandan respondents. Completion percentages in the USA dropped in the middle of each week and were strongest at the beginning and end of each trial. In Uganda, completion percentages remained high throughout the entire trial. The American students averaged 7.5 hours of sleep per night, felt well rested on 53% of days, used sleeping aids 21% of nights, and 27% of them reported caffeine use. Participants in Uganda averaged 6.4 hours of sleep per night, felt well rested over 85% of the time, none reported using a sleeping aid, and only 3% reported caffeine use.

Both pilot studies allowed us to conclude that *Magpi* is an effective and powerful tool to build a real-time geography of health data. In addition, these studies provided valuable information on reporter consistency and desirable training, as well as detecting minor technical issues that were referred, troubleshot and eliminated by *Magpi*’s technical support team.

### Feasibility mHealth study (Nodding syndrome)

We next used *Magpi* in combination with the lay operator mHealth network of eight village-based lay mHealth reporters (*vide supra*) to develop a real-time electronic map of child health, injury and illness relating to NS in northern Uganda. Surveillance data were collected for three consecutive months (August to October 2017) in a convenient sample of villages (n = 10) from two rural sub-counties (Odek in Omoro District and Awere in Pader District) heavily affected by NS (Table 1).

**Fig 1:**
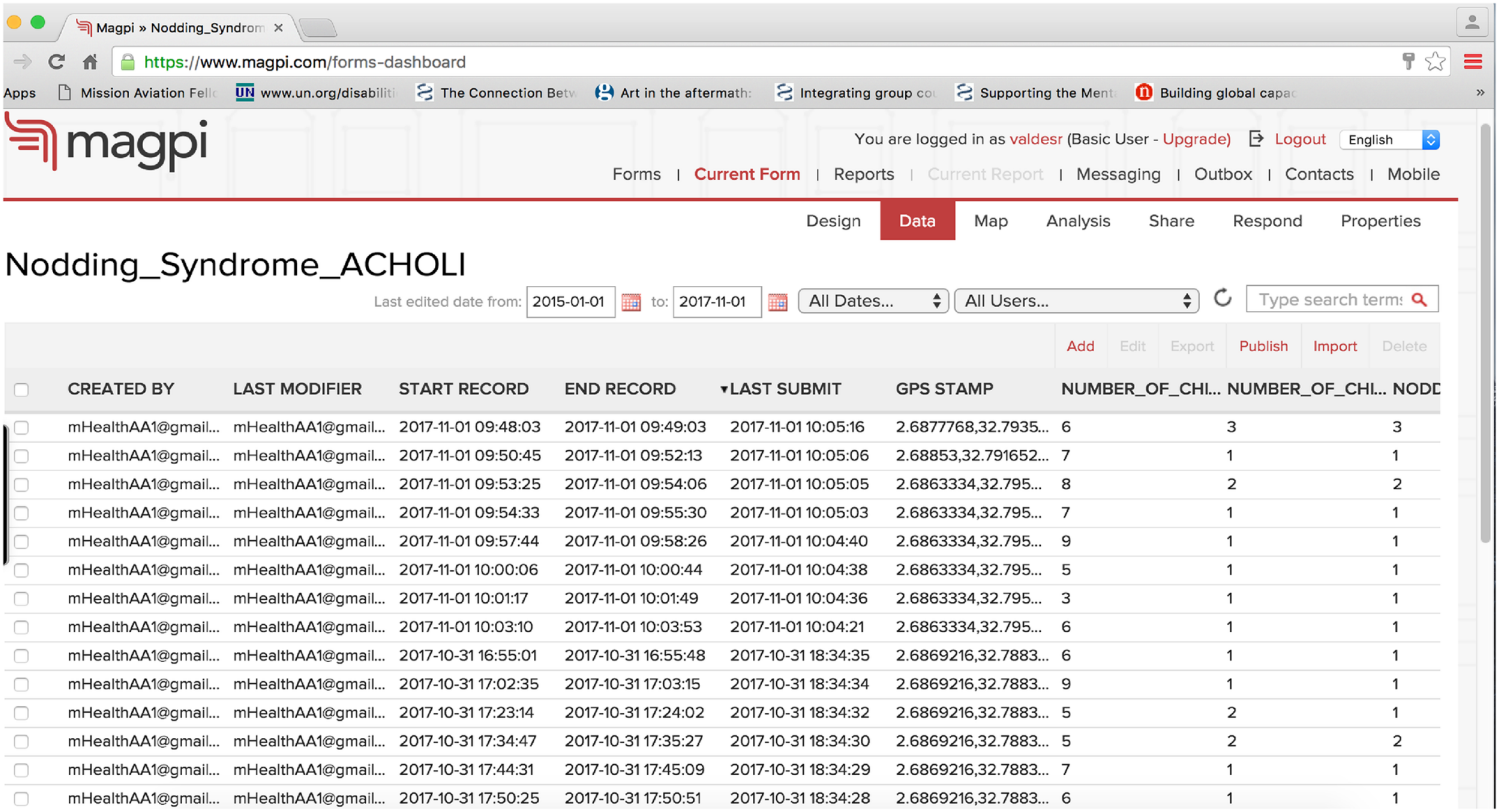
Screenshot of the *Magpi* web-interface. The image shows a) the user name associated with the mHealth reporter sending the survey record, b) the survey record start and end times, c) the time the record was submitted/uploaded to the *Magpi* server and d) the GPS stamp.

The accuracy and completeness of the surveys submitted were assessed by a) daily remote data monitoring and b) periodic quality-control visits made to a randomly selected sub-set of households. One of the major advantages of using *Magpi* was the ability to visualize, through the *Magpi* web-interface, outputs such as survey start and end times, average survey completion time, survey completion count, etc. (Fig 1). Survey quality checks were thus performed in real-time and inconsistencies were detected, reported, rectified, and cleaned in a timely manner. The monitoring potential of *Magpi* also allowed for the rapid recognition of fabricated data, based upon our expectations about the amount of time it would reasonably take to move from household to household, obtain informed consent and complete the survey. All mHealth workers were informed their movements and data authenticity could be tracked.

Almost all mHealth reporters had experience using smartphone devices prior to the surveillance feasibility project and were not intimidated by the technology. Half of them felt comfortable using the *Magpi* app within a week, and none of them found it difficult to use after a month. Despite the many implementation challenges described below, data collection proceeded in a timely and efficient manner.

### Real-time geography and monitoring of NS cases

Overall, a total of 240 households (30 households per mHealth reporter) and an average of 326 children with NS (40.7 ± 4.57 children per mHealth reporter) were monitored every week by the mHealth team. With the exception of one reporter (targeting Ludok and Olam villages) who abandoned the program on week 6 due to health-related issues, the mHealth team completed a mean of 97.3% of surveys administered per week (29.2 ± 0.63 of 30 surveys assigned per mHealth reporter).

**Fig 2:**
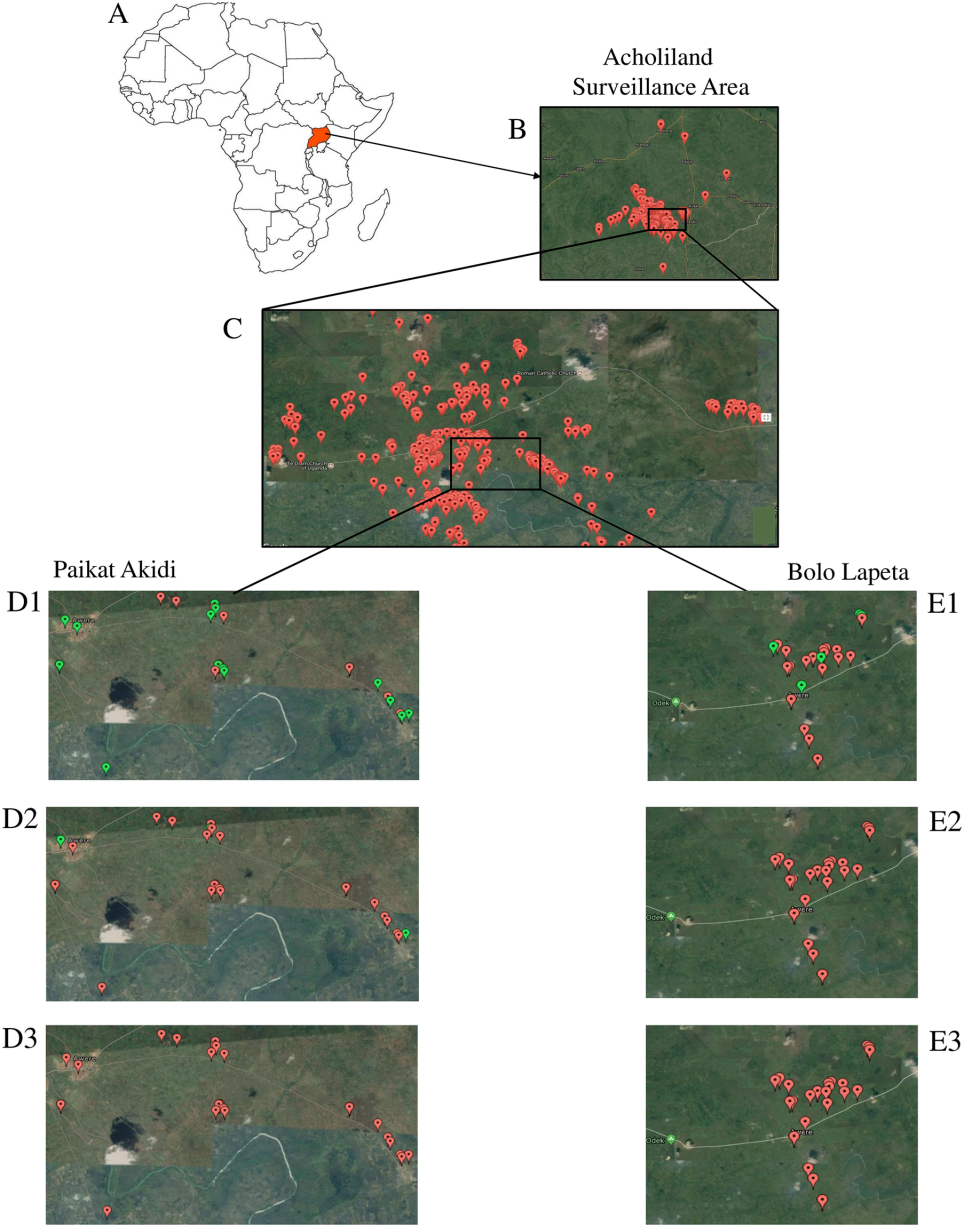
**A, B**) Geo-localized visualization of the Acholiland surveillance area. **C**) Close-up of a surveillance area section. **D, E**) Real-time electronic map of child health, injury and illness relating to NS in Paikat Akidi village (Angole parish, Awere sub-county, Pader district) and Bolo Lapeta village (Bolo parish, Awere sub-county, Pader district) at week 6 of data collection. Color coding: *Red*- Households with at least 1 child with NS; *Green*- Households with at least 1 child with NS who did not have medication (E1); was injured (E2); or died (E3) that week.

All data acquired and integrated into the *Magpi* database could be exported, with no particular conversion problems, from the platform into statistical (Excel) or spatial (*Magpi* maps) analysis software. To illustrate the system’s interoperability and quality, Fig 2 displays the surveillance area and includes a close-up of two villages (Paikat Akidi and Bolo Lapeta) at week-6 of data collection. With the map interface, it is possible to zoom in and observe the geographic distribution of NS health-related data in a particular village, and even in a specific household. For instance, the close-up in Fig 2 highlights those households with at least one child with NS that did not have anti-seizure medication (Fig 2E1, green color), was injured (Fig 2E2, green color) or died (Fig 2E3) that week.

**Fig 3:**
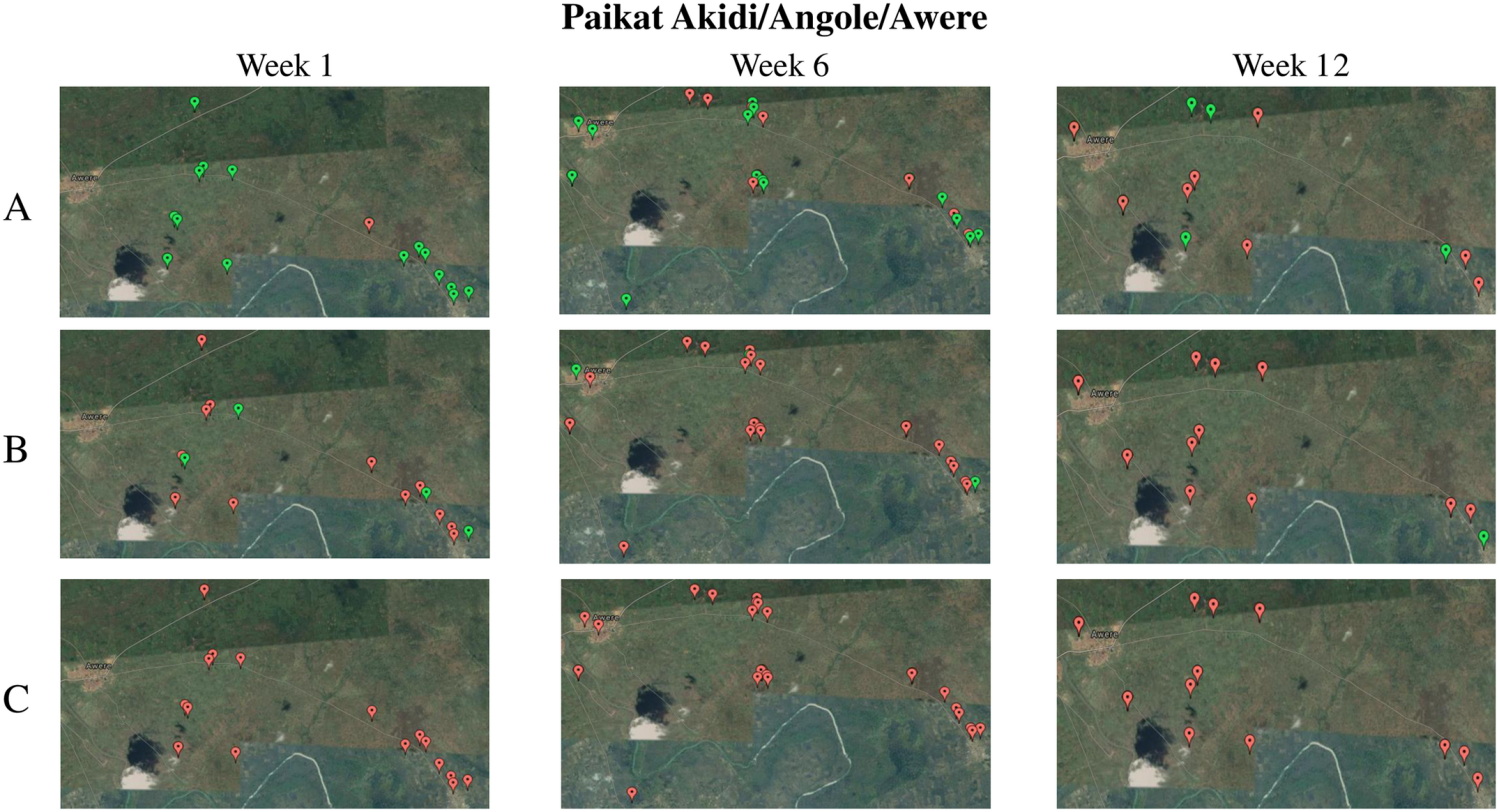
Temporal monitoring of NS health data in Paikat Akidi village, Angole parish, Awere sub-county, Pader district. **A)** Real-time electronic map for weeks 1, 6, and 12 of data collection. Color coding: *Red-* Households with at least 1 child with NS; *Green*- Households with at least 1 child with NS who did not have anti-seizure medication (A); was injured (B); or died (C) that particular week.

The *Magpi* interface also made it possible to visualize NS health-related data over a defined period of time (Fig 3) with the goal to assess fluctuations and carry out temporal interpretations. However, after comparing our maps (Fig 3) and the data collected (Table 2), it became readily apparent that many geotracers malfunctioned or, most likely, many remote areas had weak or no coverage of cellular communication, causing limitations in acquiring GPS stamps and compromising the ability to produce maps using GPS coordinates.

**Table 2:** Data collected in Paikat Akidi village (Angole parish, Awere sub-county, Pader district) throughout the 12-week study period.

Paikat Akidi/Angole/Awere												
Week	1	2	3	4	5	6	7	8	9	10	11	12
**# Households surveyed**	30	30	22	30	30	30	24	30	30	30	30	30
**# Children with NS**	45	37	22	42	37	39	40	37	29	35	41	34
**# Children with seizures**	39	39	22	41	41	43	40	39	32	42	40	42
**# Children 1st time spells**	0	0	3	0	0	0	0	0	0	1	2	0
**# Children with injuries**	14	5	3	0	2	3	4	9	2	2	0	11
**# Children that died**	0	0	0	0	0	0	0	0	0	0	0	0
**# Children w/o medication**	30	30	10	27	12	22	17	17	14	13	14	14

### Health impact assessment

While the purpose of this study was to test the feasibility of NS-related data collection and integration using *Magpi*, we used descriptive statistical analyses to assess the health impact of NS with the aim of guiding future clinical, educational and research interventions. Of the 326 affected children monitored by the mHealth team, 17 died over the 12-week study time frame and 22 siblings were reported to have nodding spells for the first time. Lack of appropriate anti-seizure medication and injuries were also registered on *Magpi* 688 times and 186 times, respectively (S1 Tables).

**Fig 4:**
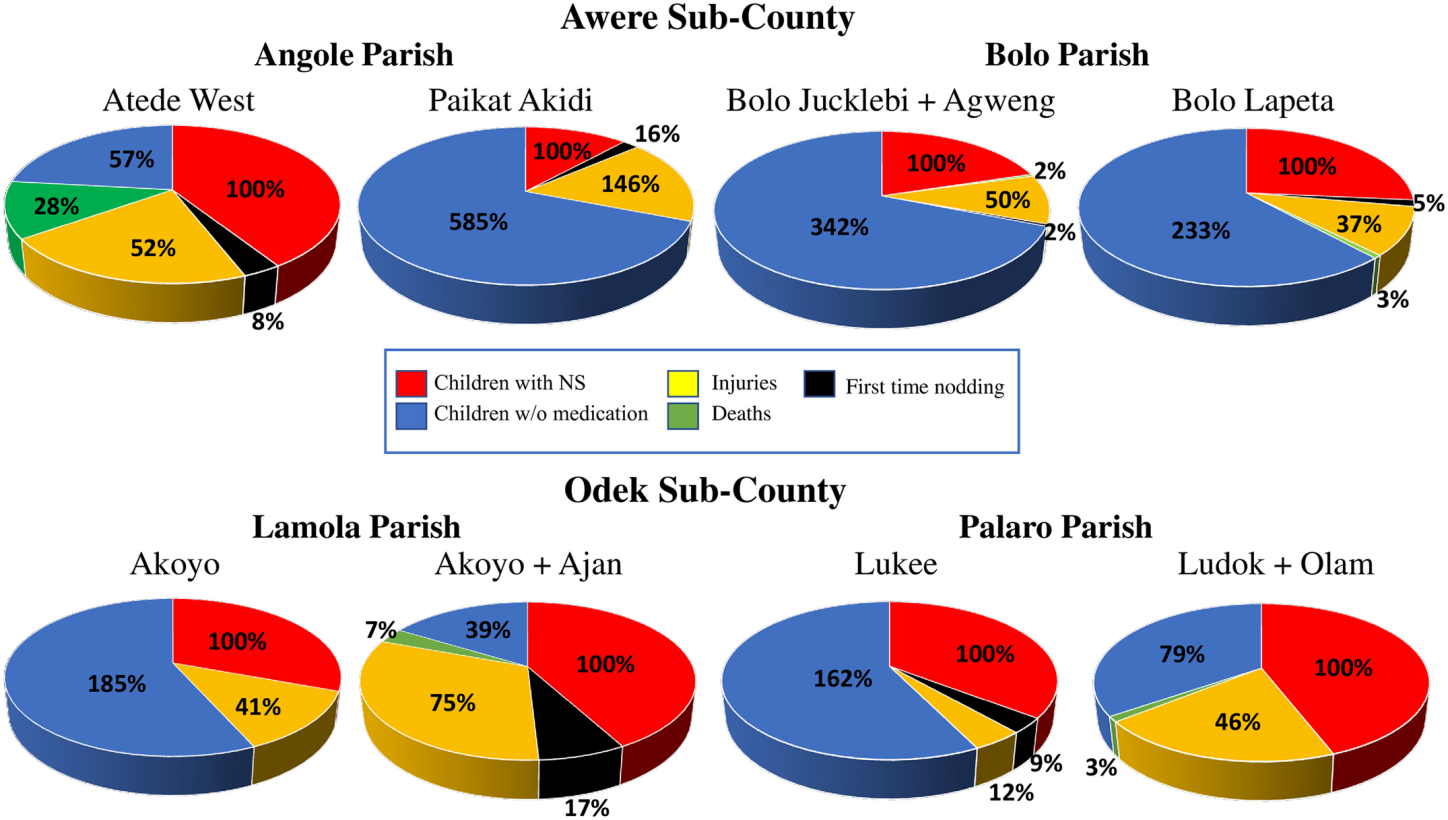
Pie charts, one for each village of study, displaying different NS health-related data across the 12-week study period. Color coding: *Red-* Proportion of children with NS; *Black-* Proportion of children with nodding spells reported for the first time; *Yellow*- Proportion of children with NS who were injured; *Green-* Proportion of children with NS who died; *Blue-* Proportion of children with NS who did not have anti-seizure medication. For comparison purposes, the number of children with NS monitored per village was normalized to 100%. Percentages above 100% are indicative of repeated outcomes. The pie chart corresponding to Ludok and Olam villages (bottom right) should be taken only for reader orientation because values summarize data collected for only 4 weeks (S1 Tables).

After normalizing the number of NS children monitored per village to 100% for comparison purposes (Fig 4, red section in each pie chart), several findings emerged. For instance, access to anti-seizure medication was extremely limited in Awere sub-county, particularly in Paikat Akidi, Bolo Juklebi/Bolo Agweng, and Bolo Lapeta, where the percentage of children without medication (Fig 4, blue section in each pie chart) was 585.1%, 341.6%, and 233.3%, respectively. We would like to highlight that the pie charts in Fig 4 display health-related information across the 12-week study and percentages above 100% are indicative of repeated (the sum of) health outcomes. For instance, the blue pie chart sections with percentages above 100% in the Paikat Akidi, Bolo Juklebi/Bolo Agweng, and Bolo Lapeta graphs denote that several children with NS in those villages did not have access to medication for a number of weeks. However, the charts do not allow the reader to discern whether, for example, 10 children did not have medication for 1 week or whether 1 child did not have medication for 10 weeks (this information is made available in the weekly S1 Tables). The goal of the pie charts was simply to provide a quick snap shot of those villages that required immediate attention. In the particular case of lack of anti-seizure medication, higher percentages indicated a greater need for medication supply.

Along the same lines, villages like Paikat Akidi and Ajan/Akoyo had the highest percentage of injured children (146.3% and 75.1%, respectively), compared to only 11.8% in Lukee. The highest mortality percentages were associated with Atede West (28.4%) and Ajan (7.3%). Ajan was also the village where respondents reported a surprisingly large percentage of children with nodding spells for the first time (16.9%), alongside Paikat Akidi (16.0%) and Lukee (8.8%). While most of these newly identified subjects proved to be longstanding NS cases in the sample given a medical evaluation, there is a possibility that the one potentially new case portends others. The possibility that the NS epidemic has not ended merits prioritized study.

While we noticed some improvement on a few health outcomes (e.g. provision of medication, reduction of injuries, clinical referrals, etc.) across the 12-week study, such progress resulted exclusively from the cooperative effort by individuals on the mHealth team (including members of the local non-profit organization *Hope for Humans*), who organized themselves to further serve their communities beyond the scope of the mHealth project.

### NS confirmation

Physical examination of the sample of newly reported and existing NS cases confirmed a diagnosis of NS. On examination, children newly reported by mHealth workers to have NS were found to have longstanding disease. Only one new case of Suspected NS was found in the sample, a boy aged 3 years with an older brother with registered NS. Around 2.5 years of age, at the sight of food, the new case developed episodes of head nodding lasting for minutes. The child was unresponsive during these periods and usually fatigued and irritable thereafter. Otherwise, on examination, he was grossly neurologically intact. The boy was registered with the Ministry of Health as a Suspected NS case and started on anti-seizure medication by a government healthcare worker. Other children that had not been registered with NS and who were identified as newly reported cases in the mHealth survey were referred to a primary health center for observation, NS confirmation and treatment.

### Challenges to implementation

The foregoing observations were made in the face of numerous technical, logistical and institutional problems that were met with innovative solutions but nevertheless substantially delayed progress. The most common technical issues were frequent power cuts, poor network coverage, slow upload speeds and unreliable GPS satellites. Inconsistent connectivity led to difficulties in survey uploading (e.g., some mHealth workers reported traveling to a specific hotspot or even climbing a particular tree to access satisfactory signal strength and network coverage). Instrument charging issues were traceable to an inability to charge phones in the field, failure to charge overnight, and short battery life of cell phones possibly exacerbated by heat. On the first day of September, all mobile phones ran out of data because the network provider failed to renew the monthly phone bundle on time. Although the surveys collected during this period were stored on the memory of the cell phone and could have been uploaded later, some reporters stopped working when they realized their data bundles were exhausted. Other reporters used hard-copy forms and entered the data in the *Magpi* app later. This last practice resulted in no data loss but large single entries (e.g., > 10 households) with only one GPS stamp. Thanks to ongoing remote monitoring, the program manager discovered there were no entries on the *Magpi* server that day and notified the problem to the field coordinator for immediate clarification. Occasional technical problems with *Magpi* software and human errors (e.g., duplicates, outliers, etc.) during daily data collection and transmission were also detected and rapidly addressed.

Some technical problems were exacerbated by logistical challenges. Some monitored households had to be replaced due to accessibility issues and poor road conditions during the rainy season. Others were replaced because of outdated household records (deceased children, relocation). A handful of householders showed some reluctance to consent and required a follow-up visit by the field supervisor. Family absences during the harvest season forced mHealth reporters to follow the subjects to their gardens. This not only slowed the process of data collection but also changed some GPS coordinates, introducing undesired variability and therefore hindering the ability to produce maps using GPS stamps. In addition, our research partner *Hope for Humans* ceased Ugandan operations on December 30^th^, 2017.

Lastly, research performed by U.S. researchers abroad poses many administrative challenges including registration, research ethics training, acquisition of research approvals from university human subjects review boards, approvals from local (Gulu), national (Uganda) and international (USA) agencies, and requirements for foreign investigators that are not user-friendly, lengthy and time-consuming. Institutional inefficiency and suboptimal cooperation consumed more than half of the 2-year grant that funded this feasibility study.

### Program-end evaluation

All mHealth workers rated the intervention, mHealth team, and outcomes very positively. The resulting gain in skills made them feel confident, enthusiastic and motivated to participate in future healthcare programs. Everyone (100%) expressed their desire to help people be healthy (e.g., as doctors, nurses, community health workers, etc.) in the future. Three of 11 (27.3%) expressed that they would also like to work in technology (e.g., cell phone manufacturing); two of 11 (18.2%) stated they would like to “sell things”; and the 2 field supervisors indicated a desire for further studies to improve their health careers (the two field supervisors have been admitted into the Lacor School of Nursing and Midwifery in Gulu). Everyone (100%) stated that money was a barrier to fulfilling their respective dreams. One mHealth reporter added that she didn’t know where to start, another expressed the lack of inadequate guidance and career mentorship, and a third added he did not have enough time, appropriate means or support from the community.

GU medical students also expressed some interest in research and future research opportunities. Healthcare providers and professionals from Gulu University, St. Mary Lacor Hospital and Awere Health Center III showed enthusiastic support for the potential of mHealth and eHealth systems (see Acknowledgments) to improve health outcomes. However, they noted funding, regulatory, and technological challenges that might hinder the integration of these approaches into primary healthcare settings.

## Discussion

### Real-time syndromic surveillance in low-income countries

One of the most remarkable examples of real-time case surveillance of neurological disease occurred during the 1991–94 Cuban epidemic of optic and peripheral neuropathy, which affected approximately 50,000 residents. Because the health system had deployed physicians across the island and required them to report unusual illnesses to the Cuban Ministry of Public Health, it was possible not only to identify the origin and track the spatial-temporal distribution and decreasing incidence of disease (from west to the east) but also to follow its clinical evolution (from pure optic to mixed and purely peripheral neuropathy forms) [16–17]. However, this extraordinary example of real-time public health surveillance is unavailable in many low-income countries, such that other solutions must be found for early disease identification and tracking.

In 1998, the African regional office of the World Health Organization (WHO) started promoting the Integrated Disease Surveillance and Response (IDSR) framework for strengthening national public health surveillance capabilities at all levels in Africa [18]. This groundbreaking program was, however, largely paper-based and has been reported to be generally inefficient and error-prone with low completeness and poor timely reporting. Considering that mobile networks have reached far more people than any other advanced communication technology in sub-Saharan Africa, data collection initiatives that use mobile phone applications to deliver life-saving information, even in the most remote and resource-poor settings, offer a new horizon for public health surveillance.

### Lay-operator mobile phone-based mHealth approaches for real-time surveillance

To date, most of the studies that have analyzed the use of mobile phones in the field of surveillance have combined the use of mobile technologies with trained staff (e.g. community health workers, nurses, midwives, health surveillance assistants, sentinel general practitioners, epidemiologist, veterinarians, pharmacists, etc.) [19–22]. In 2013, Braun and colleagues published a systematic review of mHealth tools being employed by community health workers (CHWs) in low-resource settings, mainly in Africa [23]. The findings of this review demonstrated that CHWs were using mHealth strategies with increasing effectiveness to improve delivery of maternal and child health, HIV and reproductive health services, and other general services such as immunizations and treatment of infectious diseases such as tuberculosis and malaria. In 2015, another systematic review by Agarwal and colleagues analyzed the feasibility and efficacy of mHealth strategies by frontline health workers (FHWs) as an attempt to circumvent several of the structural and systemic barriers they face in delivering health care [24]. In particular, twenty‐five studies included in this review had data collection as one of the primary mHealth functions being performed by FHWs, and several of them suggested that mobile phones are an effective way to collect and report data from the community, transfer patient‐relevant information to a centralized database and reduce the need for face‐to‐face communication between FHWs and other members of the health delivery system.

Conversely, lay health workers have been scarcely described in the literature [25] and, to our knowledge, no one has explored the use of village lay health aides as local reporters of disease. This option has the potential to support not only the early detection of disease signals (syndromic surveillance) happening within remote communities, but also near-real-time responses that might likely prevent small outbreaks from becoming large-scale emergencies. Furthermore, the lay operators in our study were stimulated by their experience with mHealth to seek additional local healthcare opportunities.

The surveillance system described herein relied on weekly inputs of neurological (NS) health data via basic smartphones operated by minimally trained lay mHealth reporters dispersed widely and resident within the communities under scrutiny. mHealth reporters needed only minimum education and experience to operate the system, with usability mostly affected by their previous level of experience with mobile phones. This approach has numerous advantages over the current paper-based documentation system, the most important of which is the accurate monitoring of events at the household level (GPS coordinates) that allows for more targeted disease control by location. Displayed on a map, the data collected provided a real-time health geography that helped identify and track disease hotspots, drug availability, injuries, deaths, and missed and new/newly reported cases of NS with no more than 1–7 days of delay. While each and every report requires medical confirmation, this is an extraordinary improvement in a region where major disease outbreaks have been recognized very slowly, monitoring is unavailable, and the distribution and utilization of vaccines and drugs are unclear. For instance, the 12-week feasibility survey identified several previously unreported longstanding cases of NS and at least one case of Suspected NS, indicating that further investigation of this epidemic is mandated. Since NS is not a recognized Neglected Tropical Disease (NTD) and, consequently NS research is poorly funded, NTD recognition and increased research funding and collaboration are needed to advance understanding of the NS epidemic in East Africa [26–27]. In addition, since the *Magpi* software supports a customized question set, the system has wide applicability for data collection across a vast array of readily recognizable health-related conditions, including dangerous infectious diseases such as EHF. Using a single system that gathers information about multiple diseases or behaviors of interest to several intervention programs may facilitate integration (at the local, regional and national level) and broad-scale surveillance and response. Collaboration among practitioners, researchers, nations, and international organizations is necessary to address the global needs of public health surveillance, provide accountability for local health status and deliver real-time early warning of potentially devastating outbreaks.

### Feasibility and limitations of the mHealth study (Nodding syndrome)

Overall, through the development, implementation, and evaluation of this syndromic surveillance system of (neurological) disease in northern Uganda, we have demonstrated the significant value and feasibility for mobile technologies to overcome health communication barriers, even in adverse weather conditions, across desperately impoverished, war-traumatized populations living in remote rural areas. Critical factors that largely influenced the success of the program included careful planning, meticulous team selection, thorough training, vigilant monitoring, supportive supervision, and effective communication between the local research team and the USA program manager. This *modus operandi* enabled problems, such as technical issues (e.g., hardware and/or software failures using the smartphones), improper handling and maintenance of equipment (e.g. inadequate cleaning/charging of solar panels), and human errors (e.g., data entry errors), to be detected and resolved as soon as they arose. In addition, we sought to create a highly motivating work environment by clearly defining roles and responsibilities (e.g., number of households to be served, frequency, geographic distance to be covered, etc.); offering fair salaries and incentives (e.g. free airtime for personal use); providing travel assistance (e.g., travel reimbursement for attended meetings, bicycles and rain gear); providing personalized certificates of excellence as a sign of appreciation; and providing recommendations for additional training and programs with the goal of building the reporters’ capacity, knowledge base, and standing in the community.

However, our study also identified several limitations. First, mobile and information communication platforms are based and dependent on technology, which is inadequately available or weak in Uganda and in many sub-Saharan African countries. Second, many barriers to implementation and sustainability limit the success of mHealth solutions beyond the pilot or feasibility stage [28–29]. In fact, a large set of the mHealth studies described in the literature are pilots and provide little or no information about the effectiveness or impact of the use of mobile technologies when included in large-scale implementations of electronic health strategies. In our particular case, full deployment may require policies that support the integration of mHealth surveillance into national strategies for health system strengthening (the potential for successful scale-up of mHealth tools increases when there is strong government ownership of the process) [30] and the commitment of local and national health agencies to invest in the cost of cell phones, cellular bundles, *Magpi* software, training, and oversight. Most importantly, because syndromic surveillance is designed to detect population patterns of disease and cannot be used to establish individual diagnoses, it is important to assess whether the healthcare workforce may have the capacity to absorb additional mobile-based responsibilities (e.g., data management and processing, clinical examination of suspect cases, deployment of clinical/educational/research interventions, etc.) before any attempt is made to scale-up.

### Etiology of Nodding syndrome

The etiology of Nodding syndrome is unknown. The medical condition was first described in Tanzania in the 1960s [31] but may have been present decades earlier [32]. In the early 1990s, an epidemic of NS impacted then-southern Sudan and, circa 1997, a second began in northern Uganda, affecting over 3000 children with a case fatality of 6.7% [33, 34]. Both epidemics were in association with civil conflict, population displacement, food shortages and malnutrition, disruption of vaccination campaigns, and lack of medical care [13,35]. Although families may have multiple children with NS [35], which raises questions of hereditable factors, the overwhelming evidence points to one or more environmental impacts that selectively prevailed during or immediately prior to the epidemic periods. Exposure to toxic food plants or environmental chemicals was ruled out in Sudan NS cases [36]. These children were shown to be more heavily infested with the nematode *Onchocerca volvulus* (OV) than village children without the neurological disease [37]. The association with OV was confirmed in Tanzania and Ugandan NS but evidence for entry of the worm into the cerebrospinal fluid or central nervous system was lacking in all cases. This led to the hypothesis that NS is an autoimmune disorder in which there was molecular mimicry between OV antigens and leiomodin-1 [38], an actin-binding protein associated with smooth muscle and present in all tissues, including relatively small amounts in brain. While leiomodin-1 antibodies were present to a greater extent in NS cases than healthy controls, they were not specific for NS and immunotherapy failed to benefit NS-affected children. Thus, instead of causing NS, the presence of nematode microfilaria may represent opportunistic infection of a host immunocompromised by malnutrition or other immunosuppressive factors [39], such as measles infection (especially in crowded displaced-person camps) and mycotoxins in moldy corn, both of which were statistically associated with NS cases versus village controls prior to onset of head nodding in the former [40]. Infantile infection with measles or rubella virus can lead to the appearance of progressive brain disorders with some similarities to NS but the search for a neurotropic virus has yet to begin. Meanwhile, recently performed electroencephalographic studies of Ugandan children with established NS have concluded that head nodding episodes are likely late-onset epileptic spasms [41], and neuropathological investigation has revealed a unique neurodegenerative disorder comparable to frontotemporal degeneration with tauopathy [42].

### Next steps

Consistent with the guiding principles of the Health Data Collaborative (https://www.healthdatacollaborative.org/what-we-do/), this study contributes to the growing body of evidence that supports the feasibility of real-time mHealth data collection on rare diseases to support local decision-making in resource-limited settings. Expanded access by local health authorities to data on NS from affected areas will increase accountability and encourage continuous improvement of data needed for unified monitoring and evaluation of access to new cases, seizure medication and deaths. In anticipation of these long-term needs, we hope to test the ability to collect full clinical details on NS cases to track disease progression and medication adherence in real time within familial clusters of NS using WOOP, an etablet application developed in Kenya for single point-of-care collection of commodities data and clinical data by lay operators to elicit protocol-driven SMS-reminders tailored to different patients by risk cohort, and thereby improve adherence to treatment in the absence of specialized medical providers.

## Supporting information

S1 Tables Survey data collected per week and per village across the 12-week study period. Coding: *Red*—Incomplete number of households (n = 30) surveyed that week. When n<30, the total number of children with NS and the total number of children with seizures were not included in the average ± SD; *Number*—Outliers or values not included in the average ± SD due to incomplete number of households surveyed or household replacement; (*)—Households replaced from one week to another due to relocation, poor road access, or death.
(DOCX)
