# Supplementary material for: A real-time medical cartography of epidemic disease (Nodding syndrome) using village-based lay mHealth reporters
Source: PLoS Negl Trop Dis. 2018 Jun 15;12(6):e0006588. doi: 10.1371/journal.pntd.0006588 (PMC6021112; doi:10.1371/journal.pntd.0006588)
Supplement: S1 Tables — Coding: Red—Incomplete number of households (n = 30) surveyed that week. When n<30, the total number of children with NS and the total number of children with seizures were not included in the average ± SD; Number—Outliers or values not included in the average ± SD due to incomplete number of households surveyed or household replacement; (*)—Households replaced from one week to another due to relocation, poor road access, or death. (DOCX) [file pntd.0006588.s001.docx]

**S1 TABLES**

| Awere/Angole/Atede West |  |  |  |  |  |  |  |  |  |  |  |  |  |
| --- | --- | --- | --- | --- | --- | --- | --- | --- | --- | --- | --- | --- | --- |
| Week | **1** | **2** | **3** | **4** | **5** | **6** | **7** | **8** | **9** | **10** | **11** | **12** | **Average + SD/Sum** |
| # Households surveyed | 30 (*) | 30 | 30 | 30 | 30 | 30 | 29 | 30 | 30 | 29 | 29 | 30 | 29.8 + 0.45 |
| # Children with NS | ~~43~~ | 39 | 38 | 38 | 39 | ~~44~~ | ~~39~~ | 39 | 39 | ~~37~~ | ~~37~~ | 39 | 38.8 + 0.46 |
| # Children with seizures | ~~29~~ | 34 | 37 | 36 | 38 | 39 | ~~38~~ | 39 | 38 | ~~36~~ | ~~36~~ | 38 | 37.4 + 1.69 |
| # Children 1st time spells | 3 | 0 | 0 | 0 | 0 | 0 | 0 | 0 | 0 | 0 | 0 | 0 | 3 |
| # Children with injuries | 10 | 3 | 2 | 2 | 3 | 0 | 0 | 0 | 0 | 0 | 0 | 0 | 20 |
| # Children that died | 9 | 2 | 0 | 0 | 0 | 0 | 0 | 0 | 0 | 0 | 0 | 0 | 11 |
| # Children w/o medication | 8 | 7 | 7 | 0 | 0 | 0 | 0 | 0 | 0 | 0 | 0 | 0 | 22 |

(*) Three households were replaced from week 1 to week 2

| Awere/Angole/Paikat Akidi |  |  |  |  |  |  |  |  |  |  |  |  |  |
| --- | --- | --- | --- | --- | --- | --- | --- | --- | --- | --- | --- | --- | --- |
| Week | **1** | **2** | **3** | **4** | **5** | **6** | **7** | **8** | **9** | **10** | **11** | **12** | **Average + SD/Sum** |
| # Households surveyed | 30 | 30 | 22 | 30 | 30 | 30 | 24 | 30 | 30 | 30 | 30 | 30 | 28.8 + 2.76 |
| # Children with NS | 45 | 37 | ~~22~~ | 42 | 37 | 39 | ~~40~~ | 37 | 29 | 35 | 41 | 34 | 37.6 + 4.50 |
| # Children with seizures | 39 | 39 | ~~22~~ | 41 | 41 | 43 | ~~40~~ | 39 | 32 | 42 | 40 | 42 | 39.8 + 3.08 |
| # Children 1st time spells | 0 | 0 | 3 | 0 | 0 | 0 | 0 | 0 | 0 | 1 | 2 | 0 | 6 |
| # Children with injuries | 14 | 5 | 3 | 0 | 2 | 3 | 4 | 9 | 2 | 2 | 0 | 11 | 55 |
| # Children that died | 0 | 0 | 0 | 0 | 0 | 0 | 0 | 0 | 0 | 0 | 0 | 0 | 0 |
| # Children w/o medication | 30 | 30 | 10 | 27 | 12 | 22 | 17 | 17 | 14 | 13 | 14 | 14 | 220 |

| Awere/Bolo/Bolo Jucklebi and Bolo Agweng |  |  |  |  |  |  |  |  |  |  |  |  |  |
| --- | --- | --- | --- | --- | --- | --- | --- | --- | --- | --- | --- | --- | --- |
| Week | **1** | **2** | **3** | **4** | **5** | **6** | **7** | **8** | **9** | **10** | **11** | **12** | **Average + SD/Sum** |
| # Households surveyed | 30 | 30 | 30 | 22 | 30 | 29 | 29 | 30 | 30 | 27 | 29 | 30 | 28.8 + 2.33 |
| # Children with NS | 50 | 49 | 49 | ~~35~~ | 49 | ~~47~~ | ~~48~~ | 49 | 49 | ~~48~~ | ~~46~~ | 47 | 48.9 + 0.90 |
| # Children with seizures | 39 | 39 | 35 | ~~25~~ | 37 | ~~36~~ | ~~36~~ | 37 | 37 | ~~42~~ | ~~36~~ | 38 | 37.4 + 1.40 |
| # Children 1st time spells | 1 | 0 | 0 | 0 | 0 | 0 | 0 | 0 | 0 | 0 | 0 | 0 | 1 |
| # Children with injuries | 3 | 2 | 1 | 5 | 3 | 3 | 4 | 1 | 0 | 0 | 1 | 1 | 24 |
| # Children that died | 0 | 0 | 0 | 0 | 0 | 0 | 0 | 0 | 0 | 0 | 0 | 1 | 1 |
| # Children w/o medication | 9 | 2 | 4 | 18 | 12 | 15 | 19 | 20 | 13 | 19 | 14 | 20 | 165 |

| Awere/Bolo/Bolo Lapeta |  |  |  |  |  |  |  |  |  |  |  |  |  |
| --- | --- | --- | --- | --- | --- | --- | --- | --- | --- | --- | --- | --- | --- |
| Week | **1** | **2** | **3** | **4** | **5** | **6** | **7** | **8** | **9** | **10** | **11** | **12** | **Average +SD/Sum** |
| # Households surveyed | 30 (*) | 28 | 28 | 30 | 30 | 30 | 30 | 30 | 30 | 30 | 30 | 30 | 29.7 + 0.78 |
| # Children with NS | ~~39~~ | ~~35~~ | ~~37~~ | 40 | 40 | 40 | 40 | 41 | 40 | 41 | 41 | 41 | 40.4 + 0.53 |
| # Children with seizures | ~~19~~ | ~~18~~ | ~~19~~ | 20 | 21 | 20 | 20 | 20 | 20 | 22 | 22 | 22 | 20.8 + 0.97 |
| # Children 1st time spells | 0 | 0 | 0 | 0 | 0 | 0 | 1 | 0 | 0 | 1 | 0 | 0 | 2 |
| # Children with injuries | 5 | 1 | 0 | 0 | 0 | 0 | 2 | 3 | 2 | 1 | 0 | 1 | 15 |
| # Children that died | 0 | 0 | 0 | 0 | 0 | 0 | 0 | 1 | 0 | 0 | 0 | 0 | 1 |
| # Children w/o medication | 14 | 10 | 7 | 6 | 7 | 8 | 9 | 5 | 4 | 7 | 9 | 8 | 94 |

(*) One household was replaced from week 1 to week 2

| Odek/Lamola/Akoyo |  |  |  |  |  |  |  |  |  |  |  |  |  |
| --- | --- | --- | --- | --- | --- | --- | --- | --- | --- | --- | --- | --- | --- |
| Week | **1** | **2** | **3** | **4** | **5** | **6** | **7** | **8** | **9** | **10** | **11** | **12** | **Average + SD/Sum** |
| # Households surveyed | 30 | 30 | 30 | 30 | 30 | 30 | 30 | 30 | 30 | 30 | 30 | 30 | 30.0 + 0.00 |
| # Children with NS | 46 | 46 | 46 | 46 | 46 | 46 | 46 | 46 | 46 | 46 | 46 | 46 | 46.0 + 0.00 |
| # Children with seizures | 20 | 20 | 20 | 19 | 17 | 17 | 17 | 17 | 18 | 17 | 17 | 18 | 18.1 + 1.31 |
| # Children 1st time spells | 0 | 0 | 0 | 0 | 0 | 0 | 0 | 0 | 0 | 0 | 0 | 0 | 0 |
| # Children with injuries | 5 | 1 | 2 | 0 | 0 | 2 | 2 | 4 | 1 | 1 | 0 | 1 | 19 |
| # Children that died | 0 | 0 | 0 | 0 | 0 | 0 | 0 | 0 | 0 | 0 | 0 | 0 | 0 |
| # Children w/o medication | 8 | 4 | 5 | 6 | 21 | 0 | 5 | 21 | 5 | 6 | 4 | 0 | 85 |

| Odek/Lamola/Akoyo and Ajan |  |  |  |  |  |  |  |  |  |  |  |  |  |
| --- | --- | --- | --- | --- | --- | --- | --- | --- | --- | --- | --- | --- | --- |
| Week | **1** | **2** | **3** | **4** | **5** | **6** | **7** | **8** | **9** | **10** | **11** | **12** | **Average+SD/Sum** |
| # Households surveyed | 0 | 29 | 29 | 30 | 30 | 30 | 30 | 30 | 30 | 30 | 23 | 30 | 29.2 + 2.10 |
| # Children with NS | - | ~~38~~ | ~~42~~ | 43 | 43 | 44 | 43 | 40 | 38 | 39 | ~~31~~ | 40 | 41.3 + 2.25 |
| # Children with seizures | - | ~~20~~ | ~~42~~ | 43 | 43 | 44 | 43 | 40 | 41 | 44 | ~~32~~ | 40 | 42.3 + 1.67 |
| # Children 1st time spells | - | 0 | 0 | 1 | 2 | 1 | 1 | 0 | 2 | 0 | 0 | 0 | 7 |
| # Children with injuries | - | 0 | 2 | 2 | 3 | 4 | 3 | 7 | 4 | 2 | 1 | 3 | 31 |
| # Children that died | - | 0 | 1 | 1 | 1 | 0 | 0 | 0 | 0 | 0 | 0 | 0 | 3 |
| # Children w/o medication | - | 9 | 5 | 1 | 0 | 1 | 0 | 0 | 0 | 0 | 0 | 0 | 16 |

| Odek/Palaro/Lukee |  |  |  |  |  |  |  |  |  |  |  |  |  |
| --- | --- | --- | --- | --- | --- | --- | --- | --- | --- | --- | --- | --- | --- |
| Week | **1** | **2** | **3** | **4** | **5** | **6** | **7** | **8** | **9** | **10** | **11** | **12** | **Average+SD/Sum** |
| # Households surveyed | 30 | 30 | 30 | 24 | 30 | 30 | 30 | 16 | 30 | 30 | 30 | 30 | 28.3 + 4.25 |
| # Children with NS | 34 | 34 | 34 | ~~26~~ | 34 | 34 | ~~46~~ | ~~18~~ | 34 | 34 | 34 | 34 | 34.0 + 0.00 |
| # Children with seizures | 33 | 34 | 34 | ~~26~~ | 33 | 34 | ~~17~~ | ~~18~~ | 34 | 34 | 34 | 34 | 33.78 + 0.44 |
| # Children 1st time spells | 1 | 0 | 1 | 0 | 0 | 1 | 0 | 0 | 0 | 0 | 0 | 0 | 3 |
| # Children with injuries | 2 | 0 | 0 | 0 | 0 | 0 | 2 | 0 | 0 | 0 | 0 | 0 | 4 |
| # Children that died | 0 | 0 | 0 | 0 | 0 | 0 | 0 | 0 | 0 | 0 | 0 | 0 | 0 |
| # Children w/o medication | 9 | 12 | 8 | 4 | 3 | 2 | 5 | 6 | 1 | 2 | 2 | 1 | 55 |

| Odek/Palaro/Ludok+Olam |  |  |  |  |  |  |  |  |  |  |  |  |  |
| --- | --- | --- | --- | --- | --- | --- | --- | --- | --- | --- | --- | --- | --- |
| Week | **1** | **2** | **3** | **4** | **5** | **6** | **7** | **8** | **9** | **10** | **11** | **12** | **Average+SD/Sum** |
| # Households surveyed | 30 (*) | 30 | 30 | 28 | 30 | 17 | 0 | 0 | 0 | 0 | 0 | 0 | 13.8 + 14.78 |
| # Children with NS | - | - | 40 | ~~35~~ | 39 | ~~23~~ | ~~-~~ | ~~-~~ | - | - | - | - | 39.5 + 0.71 |
| # Children with seizures | - | - | 0 | ~~0~~ | 0 | ~~0~~ | ~~-~~ | ~~-~~ | - | - | - | - | 0.0 + 0.00 |
| # Children 1st time spells | - | - | 0 | 0 | 0 | 0 | - | - | - | - | - | - | 0 |
| # Children with injuries | - | - | 5 | 4 | 5 | 4 | - | - | - | - | - | - | 18 |
| # Children that died | - | - | 0 | 0 | 1 | 0 | - | - | - | - | - | - | 1 |
| # Children w/o medication | - | - | 7 | 6 | 12 | 6 | - | - | - | - | - | - | 31 |

Week 1 and 2: Data not reliable

(*) Eight households were replaced from week 1 to week 2
